# Supplementary material for: Ribonuclease activity undermines immune sensing of naked extracellular RNA
Source: Cell Genom. 2025 May 6;5(5):100874. doi: 10.1016/j.xgen.2025.100874 (PMC12143340; doi:10.1016/j.xgen.2025.100874)
Supplement: Document S1. Figures S1–S11 and Tables S1–S3 [file mmc1.pdf]

Cell Genomics, Volume *5*

Supplemental information

Ribonuclease activity undermines immune sensing of naked extracellular RNA

Mauricio Castellano, Valentina Blanco, Marco Li Calzi, Bruno Costa, Kenneth Witwer, Marcelo Hill, Alfonso Cayota, Mercedes Segovia, and Juan Pablo Tosar

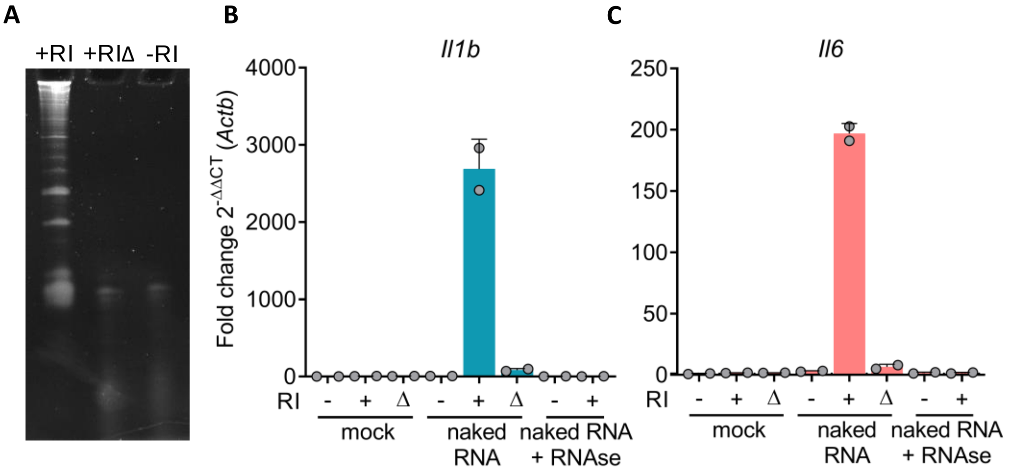

Figure S1. Naked exRNA recognition is completely dependent on RI enzymatic activity, related to Figure 1. A) Denaturing PAGE of U-2 OS total RNA incubated in 10% fetal bovine serum together with 400 U / mL RI (+RI), heat inactivated RI (+RIΔ), or without RI (-RI), for 45 minutes. B,C) *Il1b* (B) and *il6* (C) expression by RT-qPCR in BMDCs stimulated for 6 hs with 1 µg / mL naked total RNA from *E. coli* with 80 U / mL RI (+RI), heat-inactivated RI (+RIΔ), or without RI (-). RNAse1-treated *E. coli* RNA with (+RI) or without RI (-) were used as controls, as well as DPBS (mock).

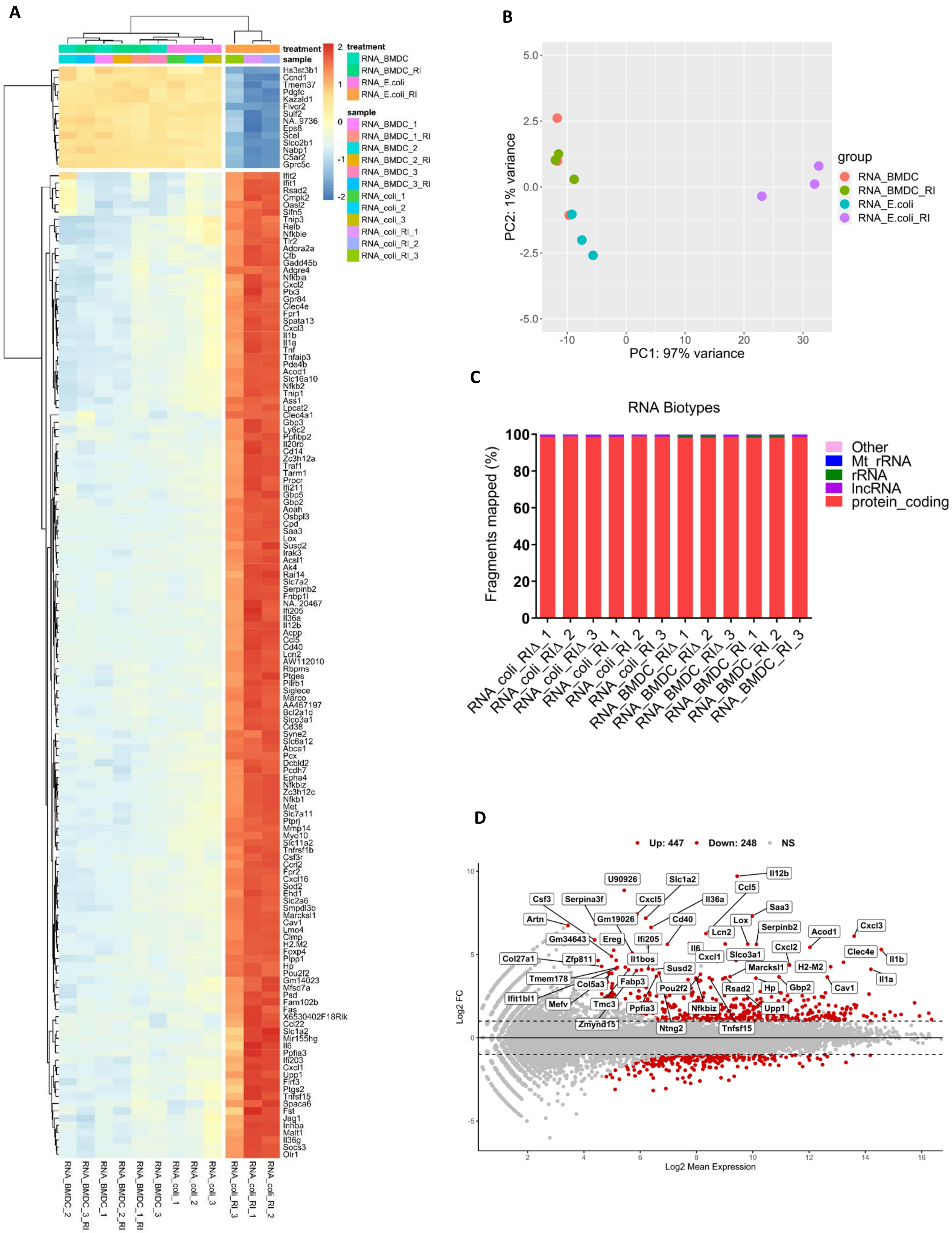

Figure S2. Additional results corresponding to the BMDC RNA-seq experiment, related to Figure 1. A) Heatmap showing top 150 most differentially regulated genes in BMDCs stimulated for 6hs with 100 ng / ml naked total RNA from *E. coli* or 100 ng / mL total RNA from BMDCs in the presence of 80 U / mL RI (+RI) or thermally inactivated RI (+RIΔ). B) PCA plots corresponding to all experimental conditions. C) Abundance of RNA biotypes across samples. D) MA-plot of BMDCs stimulated with 100 ng / mL naked total RNA (RIΔ vs. RI).

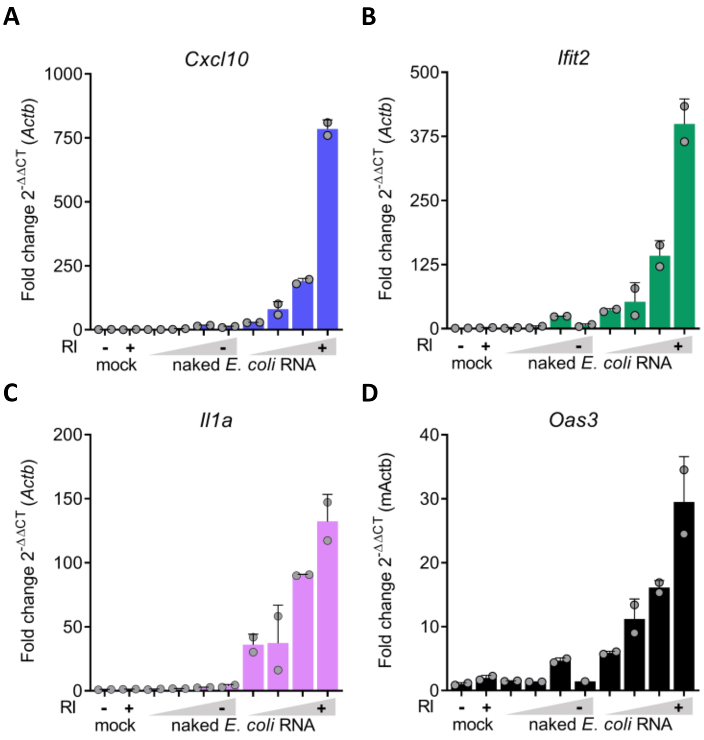

Figure S3. Induction of several pro-inflammatory cytokines and chemokines in BMDCs, related to Figure 1. Expression (RT-qPCR) of *Cxcl10* (A) *ifit2* (B) *Il1a* (C) *and Oas3* (D) in BMDCs stimulated for 6 h with varying doses (1; 5; 10; 25 µg / mL) of naked total RNA with or without 80 U / mL RI. DPBS was used as negative control.

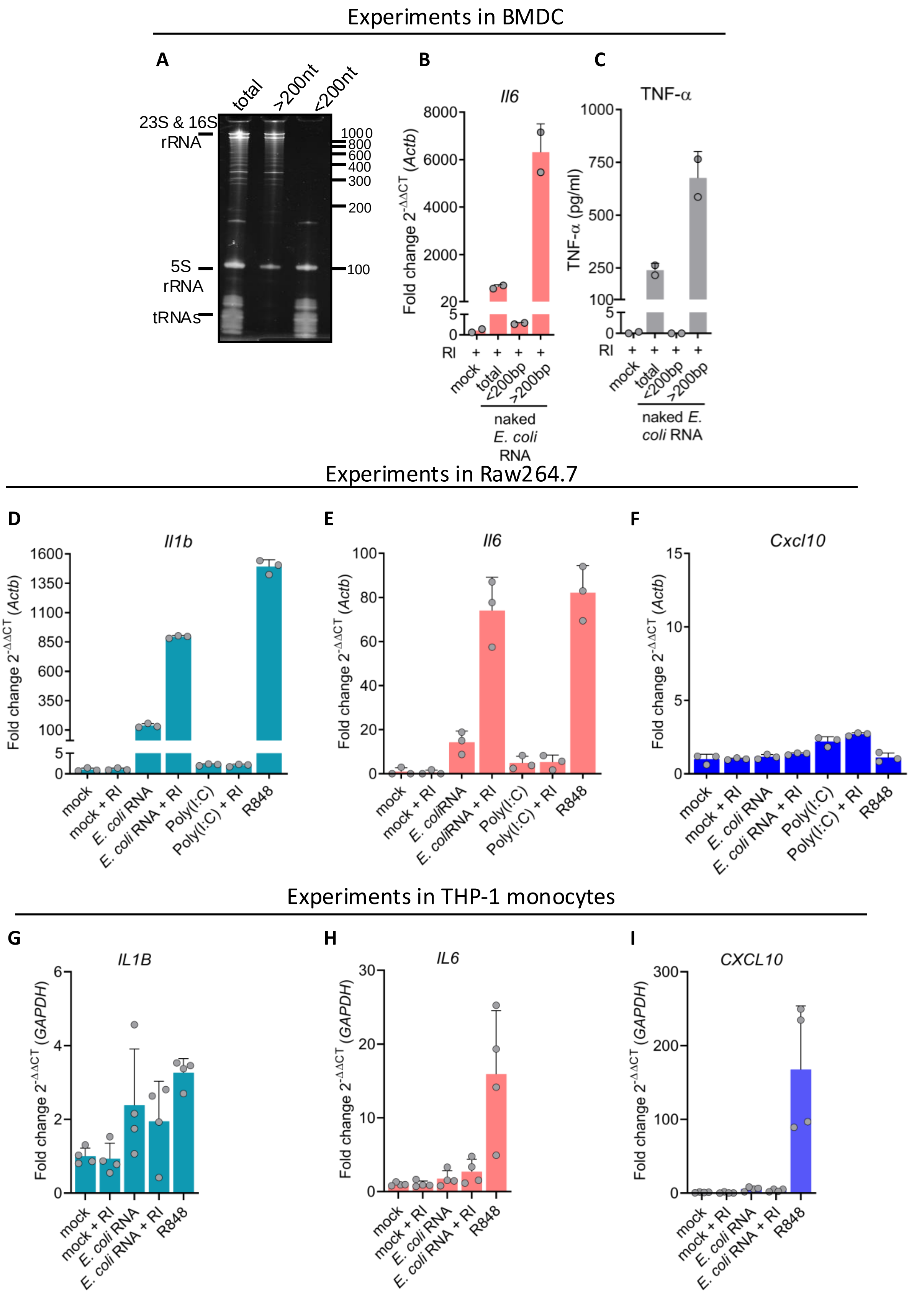

Figure S4. Response of BMDCs, Raw264.7 and THP-1 cells to different naked exRNAs, related to Figures 2 and 3. A) Denaturing PAGE of *E. coli* total RNA (total) or fractioned into "small RNAs" of less than 200 nt (< 200nt) or "large RNAs" of more than 200 nt (> 200nt). B,C) *Il6* expression (RT-qPCR) and levels of secreted TNF-α (ELISA) of BMDCs stimulated with 1 µg / mL of naked total *E. coli* RNA, "small RNAs" or "large RNAs", all in the presence of RI. Mock: DPBS. D,E,F) *Il1b* (D), *Il6* (E) and *Cxcl10* (F) expression (RT-qPCR) in Raw264.7 stimulated for 6 h with 2 µg / mL naked *E. coli* RNA, 10 µg / mL naked Poly(I:C), DMEM as mock and 1 µg / mL R848 as a positive control. When indicated, 80 U / mL RI was added. G,H,I,) *IL1B* (G), *IL6* (H) and *CXCL10* (I) expression (RT-qPCR) in THP-1 cells stimulated for 18 h with 1 µg / mL naked *E. coli* RNA, RPMI as mock and 1 µg / mL R848 as a positive control. When indicated, 80 U / mL RI was added.

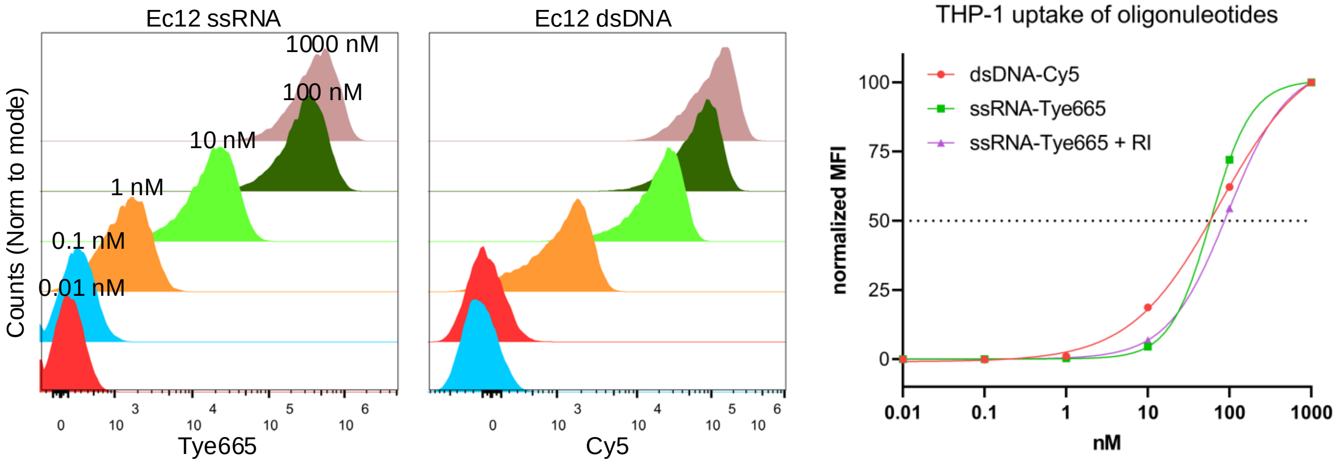

Figure S5. Uptake efficiency of Ec12 with an RNA or DNA backbone, related to Figure 3. A,B) Flow cytometry of THP-1 monocytes stimulated for 3 h with Tye665-labeled naked Ec12 ssRNA or Cy5-labeled naked Ec12 dsDNA at 0.01; 0.1; 1; 10; 100 or 1000 nM. When indicated, 160 U / mL RI was added. The histogram plots display oligonucleotide uptake at each concentration (left and center panels). A curve representing the normalized mean fluorescence intensity (MFI) is plotted against the concentration for each oligonucleotide (right panel). Data was fitted to sigmoidal curves, and the EC50 values were determined.

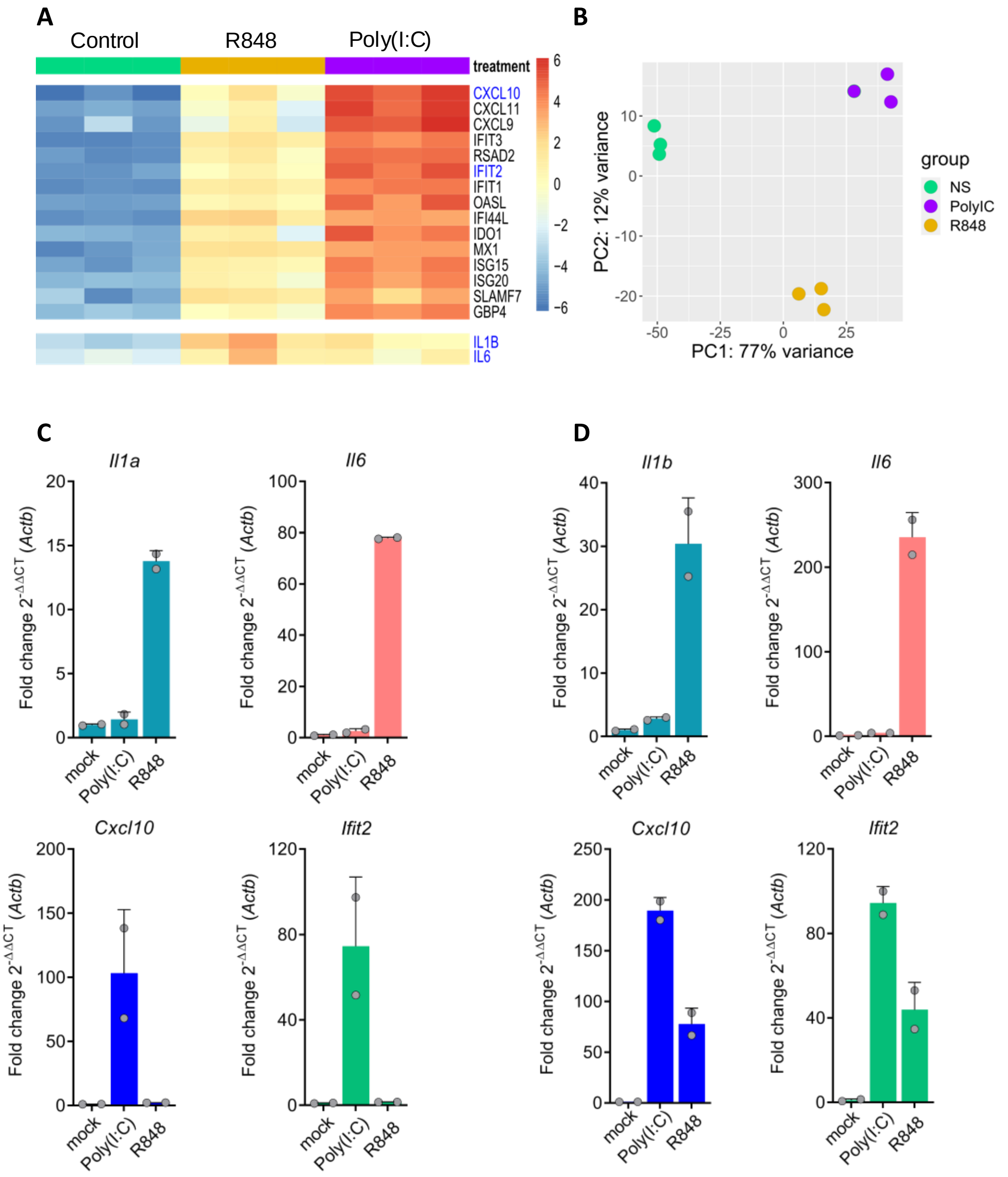

Figure S6. Transcriptional signature of Mo-DCs and JawsII dendritic cell line in response to R848 and poly(I:C), related to Figure 3. A,B) Data mining and differential gene expression analysis of a transcriptomic dataset[S1] (GEO:GSE125817) from monocyte derived dendritic cells stimulated with Poly(I:C) or R848 for 6 h. A, B) Heatmap (A) with the top 15 most upregulated genes (including *Il1b* and *Il6*) and the corresponding PCA (B) plot. C,D) Expression (RT-qPCR) of *Il1b (or Il1a), il6, Cxcl10 and Ifit2* in JawsII dendritic cell line (C) or in BMDCs (D), stimulated for 6 h with 10 µg / mL poly(I:C) or 100 ng / mL R848. DPBS was used as negative control.

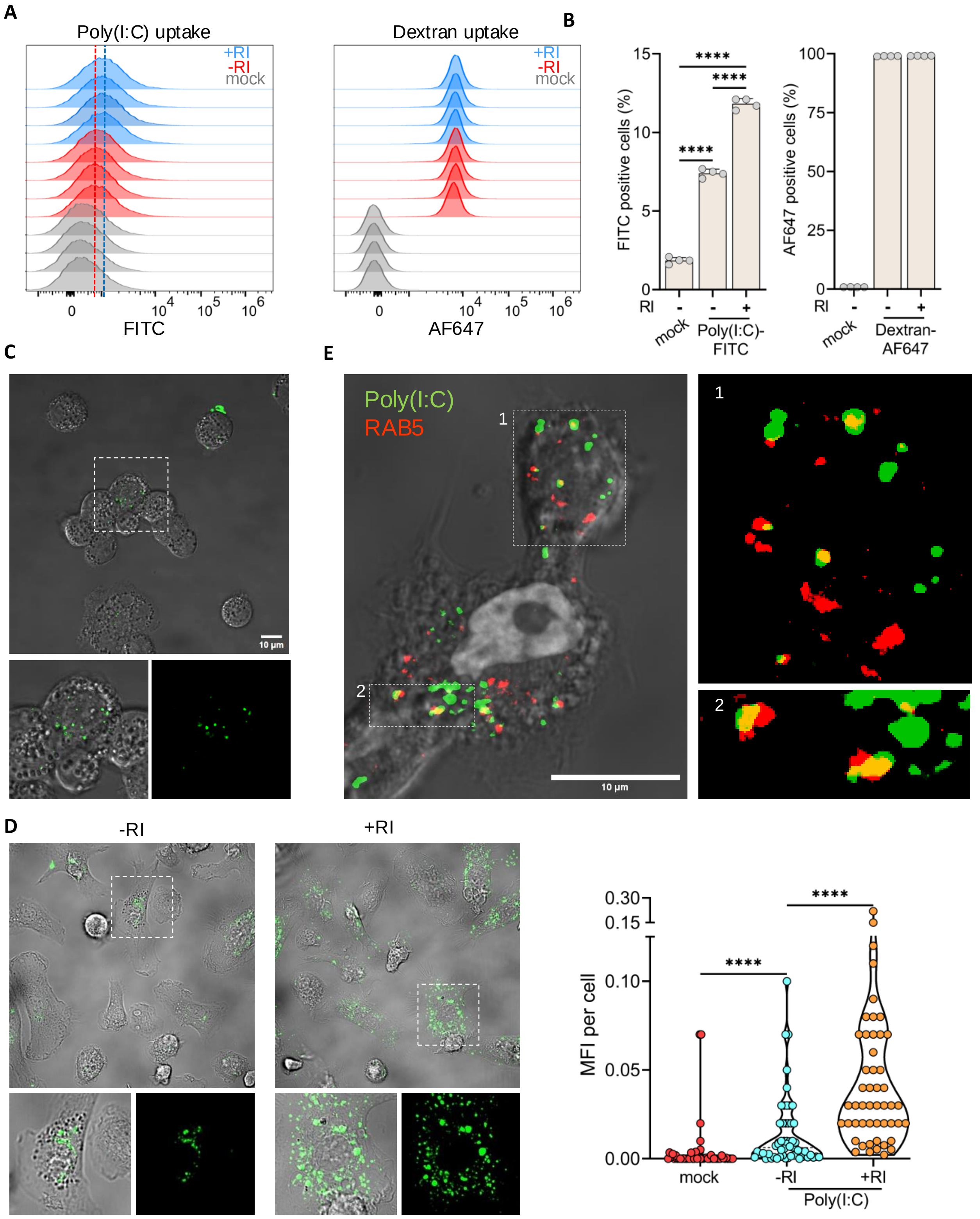

Figure S7. Uptake of labeled dsRNA by THP-1 and BMDCs, related to Figure 3. A,B) Flow cytometry of THP-1 monocytes stimulated for 3 h with naked fluorescein-labeled poly(I:C) at 0.5 µg / mL or AF647-Dextran, with or without 160 U / mL RI. Histograms (A) and bar plots (B) with the percentage of positive cells are shown. C,D) Confocal microscopy of THP-1 monocytes (C) or BMDCs (D) stimulated for 1 h with naked fluorescein-labeled poly(I:C) at 0,5 µg / mL. For BMDCs, experiments were carried out with or without 80 U / mL RI. E) Confocal microscopy of THP-1 macrophages stimulated for 30 min with naked fluorescein-labeled poly(I:C) at 0,5 µg / mL. Nuclei (white), dsRNA (green) and RAB5 (red) are visualized. Colocalization of dsRNA with RAB5 is depicted in yellow in the merged image. **** $p < 0.001$; Kruskal-Wallis with Dunn`s test.

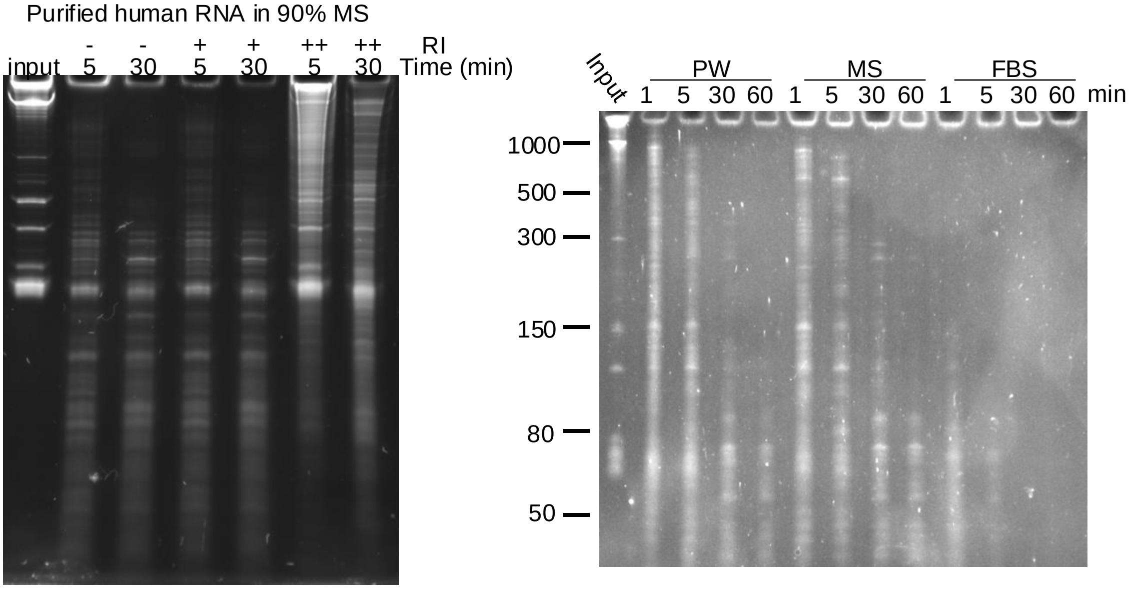

Figure S8. RNA stability in different fluids assessed by PAGE, related to Figure 5 and 6. A) Denaturing PAGE of human total RNA incubated in 90% mouse serum for either 5 or 30 min in the presence of 160 U / mL (++), 40 U / mL (+), or without RI (-). B) RNA stability assays in different biofluids. Denaturing PAGE of U-2 OS RNA incubated ex vivo in peritoneal wash (PW), mouse serum (MS) or fetal bovine serum (FBS). RNA degradation was analyzed after 1, 5, 30 or 60 min. All biofluids were diluted to 3.33 % (v/v) in DPBS.

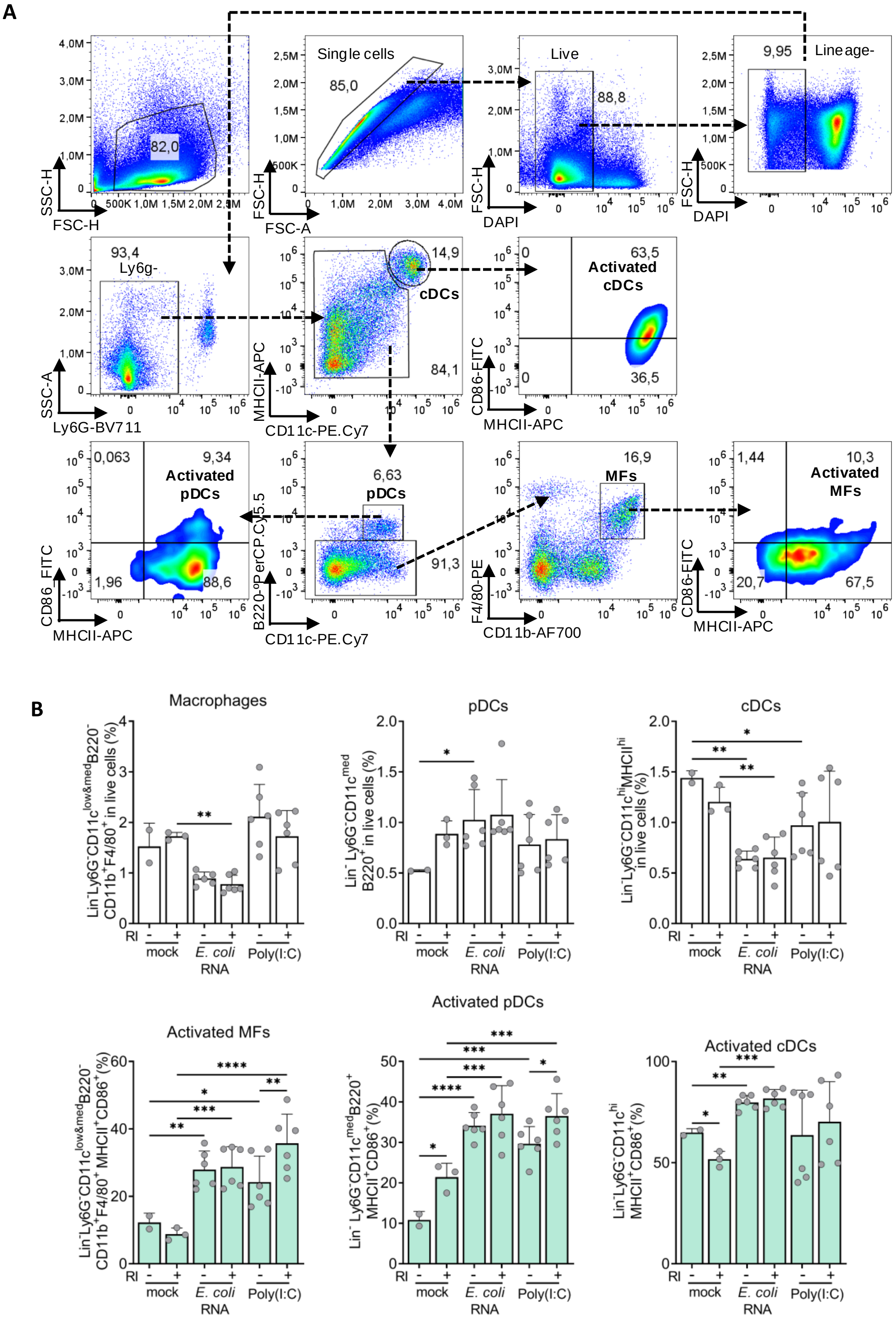

Figure S9. Gating strategy and the effect of naked exRNAs on splenic myeloid cells, related to Figure 5. A) Flow cytometry gating strategy to study splenic cDC, pDC and macrophages after intravenous administration of naked RNA stimuli. Percentages correspond to the parental gates. B) Flow cytometry analysis of spleen cells 6 h after intravenous administration of either 200ng naked Poly(I:C), or 10 µg of naked total *E. coli* RNA, with or without 480 U RI. PBS was used as mock. The percentage of total macrophages (Lin$^-$Ly6G$^-$CD11c$^{low\&med}$B220$^-$ CD11b$^+$F4/80$^+$), pDCs (Lin$^-$ Ly6G$^-$CD11c^{med}B220$^+$), cDCs (Lin$^-$Ly6G$^-$CD11c^{hi}MHCII^{hi}), gated in live cells is shown in top panels. The percentage of activated macrophages, pDCs, cDCs, (defined as MHCII$^+$CD86$^+$) gated under their corresponding cell type is shown in bottom panels with bar plots shaded in green. $*p < 0.05$, $**p < 0.01$, $***p < 0.001$; $****p < 0.0001$; one-way ANOVA with Fisher's test (if data had normal distribution and variance homogeneity) or Welch ANOVA (if data had normal distribution but lacked variance homogeneity).

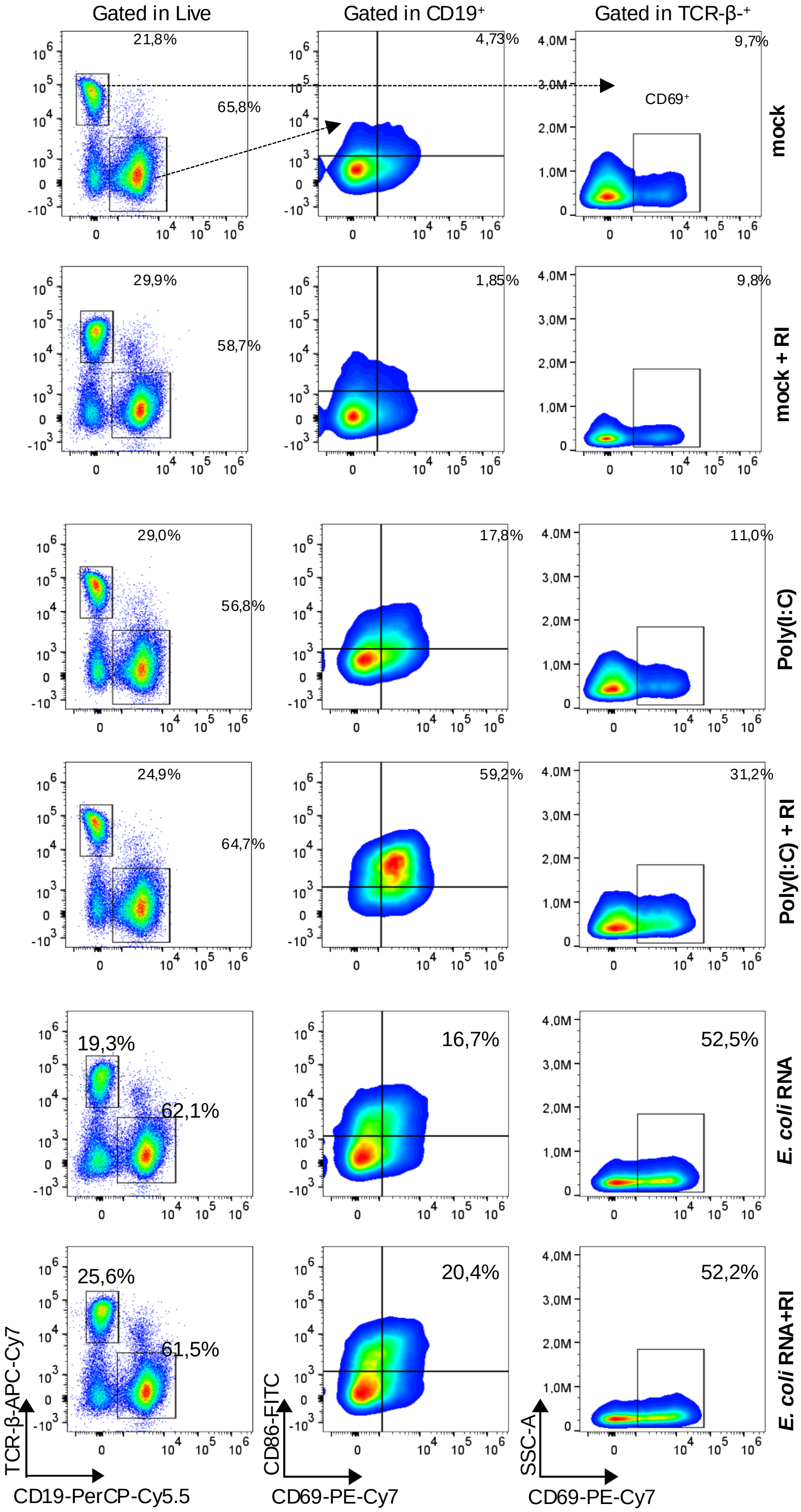

Figure S10. Effect of naked exRNA on splenic lymphoid cells, related to Figure 5. A) Flow cytometry gating strategy to study splenic B and T cells populations after intravenous administration of RNA. B) Dot plots showing flow cytometry analysis of spleen cells 6 h after intravenous administration of 10 μg naked total *E. coli* RNA, 1 μg naked poly(I:C), or DPBS, with or without 480 U RI. Percentages correspond to the parental gates.

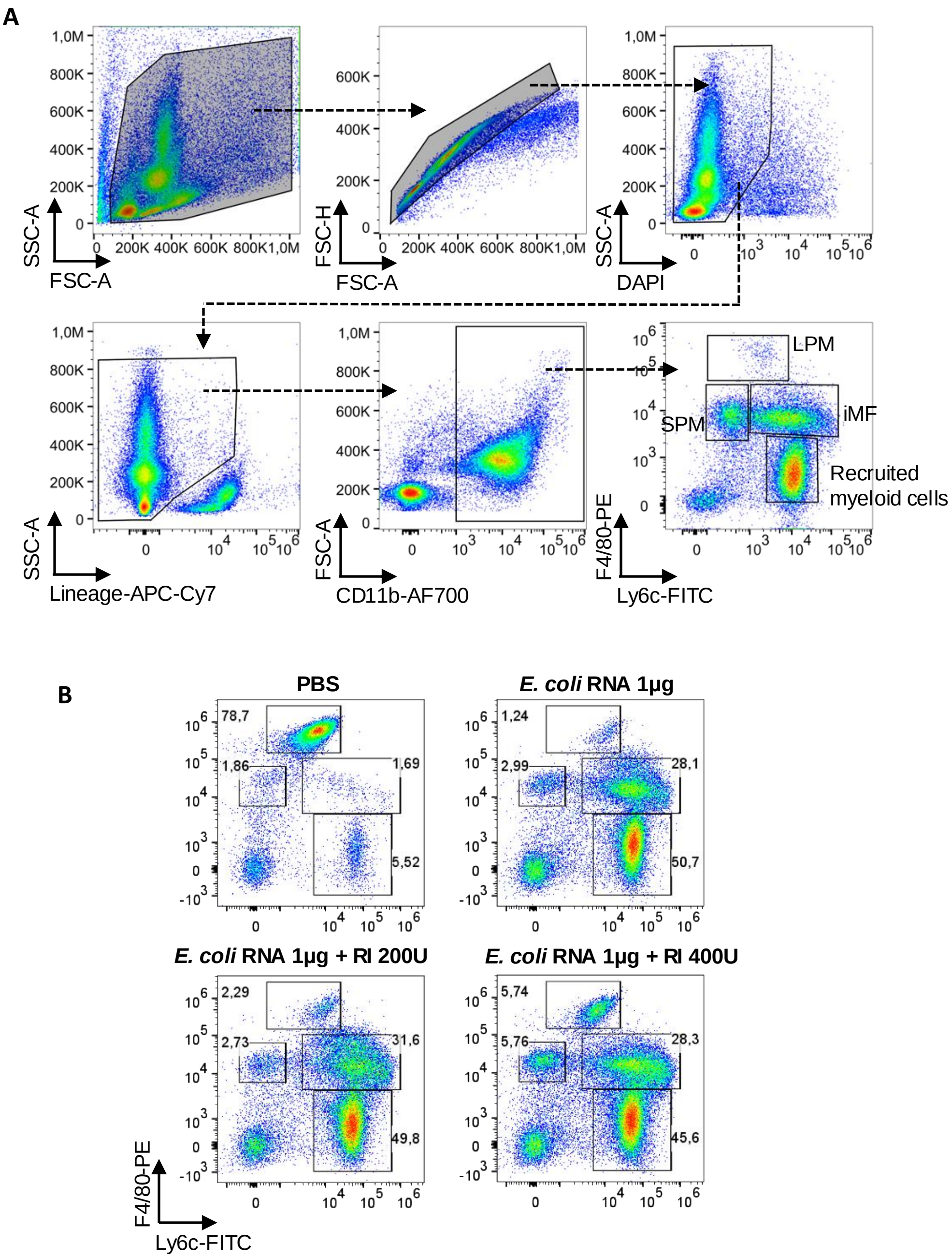

Figure S11. Gating strategy to study mice peritoneal cells, related to Figure 6. A) Gating strategy used for analyzing resident large (LPM) and small (SPM) peritoneal macrophages, recruited myeloid cells, and inflammatory macrophages (iMF) by flow cytometry. Cells were analyzed 24 hours after i.p. administration of 12.5 µg of total naked *E. coli* RNA. B) Peritoneal cell populations gated on Lineage-CD11b+ cells, 24 h after administration of DPBS or 1 µg of naked *E. coli* RNA with the indicated RI doses. Cell percentages are based on the parental gates.

Table S1. Primers for RT-qPCR, related to Star Methods		
Gene	Forward Primer	Reverse Primer
murine *Il6*	5'-AAGCCAGAGTCCTTCAGAGAG-3'	5'-GTCTTGGTCCTTAGCCACTCC-3'
murine *Il1a*	5'-AGTCAACTCATTGGCGCTTG-3'	5'-AGAGAGAGATGGTCAATGGCAG-3'
murine *Cxcl10*	5'-AGTGCTGCCGTCATTTTCTG-3'	5'-GCAGGGATGATTTCAAGCTTCC-3'
murine *Ifit2*	5'-TGCTTTGAGCGCTTTGACAC-3'	5'-TCGCAGATTGCTCTCCAGTG-3'
murine *Oas3*	5'-AGGCTACCGTGTACGCATC-3'	5'-CTTCACACAGCGGCCTTTACC-3'
murine *Il1b*	5'-TGCCACCTTTTGACAGTGATG-3'	5'-ATGTGCTGCTGCGAGATTTG-3'
murine *Actb*	5'-TGGCTCCTAGCACCATGAAG-3'	5'-AACGCAGCTCAGTAACAGTCC-3'
NanoLuc	5'-ATGGTCTTCACACTCGAAGATTTCG-3'	5'-CTGGACACACCTCCCTGTTC-3'
human *IL6*	5'-CCTGAACCTTCCAAAGATGGC-3'	5'-TTCACCAGGCAAGTCTCCTCA-3'
human *IL1B*	5'-ATGATGGCTTATTACAGTGGCAA-3'	5'-GTCGGAGATTCGTAGCTGGA-3'
human *CXCL10*	5'-GTGGCATTCAAGGAGTACCTC-3'	5'-TGATGGCCTTCGATTCTGGATT-3'
human *GAPDH*	5'-CGGAGTCAACGGATTTGGTC-3'	5'-TTCCCGTTCTCAGCCTTGAC-3'

Table S2. Primers for in vitro transcription template amplification, related to Star Methods

Gene	Forward Primer	Reverse Primer
NanoLuc	5'-ACGACGTAATACGACTCACTATAGG GTATCCGCCACCATGGTCTTCACACTCG-3'	5'-TTTTTTTTTTTTTTTTTTTTTTTTTTTTTT CGACTCTAGAATTATTACGCCAGAATG-3'
eGFP	5'- ACGACGTAATACGACTCACTATAGGGTATC CGCCACCATGGTGAGC-3'	5'-TTTTTTTTTTTTTTTTTTTTTTTTTTTTTTT ACTTGTACAGCTCGTCCATGC-3'

Table S3. Antibodies mixes used, related to Star Methods		
.	Antibodies	Concentration
BMDCs	CD40-FITC	1/500
	CD86-PE	1/200
	MHCII-APC	1/200
Spleen myeloid cells	CD19-APC-Cy7	1/200
	TCR-B-APC-Cy7	1/200
	Ly6G-BV711	1/200
	CD11c PE-Cy7	1/200
	MHC-II-APC	1/200
	B220-PerCP-Cy5.5	1/200
	F4/80-PE	1/200
	CD11b-AF700	1/200
	CD86-FITC	1/500
Spleen lymphoid cells	CD19-PerCP-Cy5.5	1/200
	TCR-B-APC-Cy7	1/200
	CD69-PECy7	1/200
	CD86-FITC	1/500
Peritoneal macrophages and monocytes	CD19-APC-Cy7	1/200
	TCR-B-APC-Cy7	1/200
	CD11b-AF700	1/200
	F4/80-PE	1/200
	Ly6c-FITC	1/500

Supplemental references

[S1] Johnson, J.S., De Veaux, N., Rives, A.W., Lahaye, X., Lucas, S.Y., Perot, B.P., Luka, M., Garcia-Paredes, V., Amon, L.M., Watters, A., et al. (2020). A Comprehensive Map of the Monocyte-Derived Dendritic Cell Transcriptional Network Engaged upon Innate Sensing of HIV. Cell Rep *30*, 914–931. https://doi.org/10.1016/j.celrep.2019.12.054.
